# Is Previous Upper Abdominal Surgery a Contraindication for Laparoscopic Cholecystectomy?

**DOI:** 10.7759/cureus.14272

**Published:** 2021-04-03

**Authors:** Mehmet Kağan Katar, Pamir Eren Ersoy

**Affiliations:** 1 General Surgery, Yozgat Bozok University Faculty of Medicine, Yozgat, TUR

**Keywords:** laporoscopic cholecystectomy, cholelithiasis, abdominal surgery, adhesions

## Abstract

Background and objective

In this era of minimally invasive surgery and enhanced recovery procedures, laparoscopic cholecystectomy (LC) is the prevailing treatment method for symptomatic cholelithiasis. However, there are some contraindications for this operation, such as a previous upper abdominal surgery. Additionally, the median conversion rate of LC is 5%. In this study, we aimed to investigate the effect of previous upper abdominal surgery on LC.

Methods

The study was designed as a single-center, retrospective, and observational analysis. A total of 277 LC patients were evaluated by classifying them into two groups - group A: those without previous upper abdominal surgery; group B: those with a history of previous upper abdominal surgery.

Results

Not surprisingly, the operation time and the degree of adhesions in group B were significantly higher compared to group A (p<0.001). On the other hand, there were no significant differences between the two groups in terms of complication rates, conversion rates, and the length of hospital stay (p=0.118, p=0.761, p=0.083, respectively).

Conclusion

LC is a safe method for cholelithiasis even in patients with a history of upper abdominal surgery. Previous upper abdominal surgery does not affect the conversion rates and length of hospital stay. Hence, previous upper abdominal surgery should not be accepted as a contraindication for LC.

## Introduction

Currently, the standard treatment for symptomatic cholelithiasis is laparoscopic cholecystectomy (LC). LC has some advantages over the open approach, including better cosmetic outcomes, less postoperative pain, and shorter hospital stay [1]. On the other hand, it has been reported that the rate of conversion of LC to open surgery is around 8.9% in general and higher than that in acute cholecystitis cases in particular compared to elective cases [2].

In the early years of LC, pregnancy, previous abdominal surgery, obesity, cirrhosis, and acute cholecystitis were accepted as absolute contraindications for the laparoscopic technique. By the early 2000s, with the increase in laparoscopic experience among surgeons and the advancement in laparoscopy technology, previous upper abdominal surgery in patients came to be regarded as only a relative contraindication for LC [3,4]. Nowadays, based on the further progress in experience and technology, we think that the discussion about LC in patients with a history of previous upper abdominal surgery should be revisited. Therefore, in this study, we aimed to investigate the effect of previous upper abdominal surgery on LC.

## Materials and methods

The Clinical Research Ethics Committee at our university approved our study. The study was designed as a single-center, retrospective, and observational analysis and was conducted in accordance with the Declaration of Helsinki.

Surgeons at our clinic retrospectively analyzed the data of all patients who underwent LC between January 2017 and July 2020 from the hospital data registry system. The inclusion criteria in our study were as follows: patients over the age of 18 years who underwent LC (emergency or elective), and whose medical data records were accessible. Meanwhile, the exclusion criteria were pregnancy, undergoing another invasive procedure in addition to LC (such as sleeve gastrectomy), single port laparoscopic and mini-LC procedures, and previous upper abdominal surgery due to malignancy. A total of 277 patients were included in the study. The patients who did not have a history of upper abdominal surgery were classified as group A, while those who did were classified as group B.

All patients were evaluated in terms of demographic data, surgery type, American Society of Anesthesiologists (ASA) score [5], operation time, intra-abdominal adhesion score, the rate of conversion to open surgery, length of hospital stay, and early (<30 days) complication rates. Intra-abdominal adhesions were defined according to the Blauer and Collins score as follows - grade 0: no adhesions; grade 1: thin, narrow, and easily separable adhesions; grade 2: thick adhesions in a well-defined area; grade 3: thick and widespread adhesions in a well-defined area; and grade 4: thick and widespread adhesions, including adhesions to the anterior and posterior abdominal wall [6].

Traditional four-port LC was applied to all patients included in the study. In patients with midline incision scar, pneumoperitoneum was achieved by using the open technique (the Hasson technique) at the umbilicus level [7]. If adhesions were found under the umbilical incision, these were bluntly finger-dissected. A Veress needle was used to achieve pneumoperitoneum in the rest of the patients. A 10-mm trocar at the umbilicus level, a 10-mm trocar under the xiphoid, and a 5-mm trocar in the subcostal area in the midclavicular line and in the anterior axillary line were used. After entering the peritoneal cavity, adhesions were separated in order to visualize the operation area and perform the operation. First, the cystic duct and cystic artery were seen by dissecting the Calot's triangle. It was cut off with a clip. Then the gall bladder was separated from the liver.

Statistical analysis

The IBM SPSS Statistics v22.0 (IBM, Armonk, NY) program was used for the statistical analysis of the data. The Kolmogorov-Smirnov test was used to test whether the distribution was normal. The Chi-square test was employed to compare groups pertaining to categorical variables. The comparison of the two groups was done with the t-test for parametric data and the Mann-Whitney U test for non-parametric data. A p-value of <0.05 was considered statistically significant.

## Results

While 235 of the patients included in our study did not have a history of upper abdominal surgery (group A), 42 patients had previously undergone such surgeries (group B) for various reasons (eight patients: sharp abdominal trauma; 10 patients: blunt abdominal trauma; 14 patients: peptic ulcer perforation; six patients: various bowel operations; and four patients: liver hydatid cyst). The mean age of the patients in groups A and B was 46.17 ±13.23 and 49.50 ±11.49 years, respectively, and there was no significant difference in age among the groups (p=0.098). In addition, while the percentage of females was 51.9% in group A, it was 42.9% in group B; however, this difference was also not statistically significant (p=0.280). Moreover, there was no significant difference between the two groups in terms of body mass index (BMI) and ASA score (p=0.073, p=0.639, respectively). The number of patients taken for the emergency operation was 68 (28.9%) in group A and 16 (38.1%) in group B. There was no difference between the two groups in terms of operation type (p=0.234). Sociodemographic and preoperative data of the patients are given in Table 1.

**Table 1:** Patients’ sociodemographic and preoperative data SD: standard deviation; BMI: body mass index; ASA: American Society of Anesthesiologists

Variables			Group A (n=235)	Group B (n=42)	P-value
Age, years, mean ±SD			46.17 ±13.23	49.50 ±11.49	0.098
Gender					
	Female, n (%)		122 (51.9)	18 (42.9)	0.28
	Male, n (%)		113 (48.1)	24 (57.1)	
BMI, kg/m², mean ±SD			27.10 ±5.05	28.48 ±4.62	0.073
ASA score					
		I, n (%)	24 (10.2)	3 (7.1)	0.639
		II, n (%)	187 (79.6)	33 (78.6)	
		III, n (%)	24 (10.2)	6 (14.3)	
Surgery type					
		Elective, n (%)	167 (71.1)	26 (61.9)	0.234
		Emergency, n (%)	68 (28.9)	16 (38.1)	

When the two groups were compared in terms of the operation time, the mean operation time in group B was significantly higher compared to group A (67.02 ±21.24 vs. 43.19 ±10.17 minutes) (p<0.001). In addition, the adhesion score was also significantly higher in group B (p<0.001). The rates of conversion of laparoscopic surgery to open surgery were similar in both groups (p=0.761). Three patients (1.3%) in group A developed early complications (bile duct injury in two patients and wound infection in one patient), while two patients (4.8%) in group B developed such complications (bile duct injury in one patient and wound infection in one patient). Although the early complication rate was higher in group B, this difference was not significant (p=0.118). In addition, the average length of stay in the hospital was 2.17 ±3.10 days in group B, while it was 1.50 ±1.20 days in group A, and no statistical difference was found between the groups (p=0.083). The intraoperative and postoperative data of the patients are shown in Table 2. Of note, no patient in either group died within 30 days of the operation.

**Table 2:** Patients' intraoperative and postoperative data SD: standard deviation

Variables		Group A	Group B	P-value
Operation time, minutes, mean ±SD		43.19 ±10.17	67.02 ±21.24	<0.001
Adhesion score				
	Grade 0, n (%)	201 (85.5)	-	<0.001
	Grade 1, n (%)	26 (11.1)	2 (4.8)	
	Grade 2, n (%)	8 (3.4)	6 (14.3)	
	Grade 3, n (%)	-	16 (38.1)	
	Grade 4, n (%)	-	18 (42.9)	
Conversion to open surgery, n (%)		4 (1.7)	1 (2.4)	0.761
Early complication, n (%)		3 (1.3)	2 (4.8)	0.118
Length of hospital stay, days, mean ±SD		1.50 ±1.20	2.17 ±3.10	0.083

## Discussion

In our study, we found that the intra-abdominal adhesion score was higher in patients who had previous upper abdominal surgery compared to those who did not. This was an expected result and aligns with previous studies [8]. We also found that the mean operation time was longer in patients with a history of upper abdominal surgery compared to those without. We think that the higher rate of intra-abdominal adhesion in those who had previous upper abdominal surgery can be explained by the fact that the surgical adhesiolysis takes more type in such patients and thus prolongs the operation time. Another reason for longer operation time may have been the longer duration of time spent for the insertion of the first trocar. We preferred to place the first trocar with the open approach in all patients who had previous upper abdominal surgery. This may have prolonged the operation time. At first glance, the prolonged operation time, and therefore the duration of anesthesia, seems to be a disadvantage for the patient. However, as long as the increase in time does not reach extreme levels, the advantages of laparoscopic surgery outweigh the drawbacks. As a result of lowering the level of metabolic trauma and shortening the recovery period, the patient’s injury is minimized, and discharge and return to work are accelerated. In addition to the medical gains, a significant economic gain can also be achieved by shortening the duration of hospital stay and decreasing the time spent away from work [9].

In this study, the rates of conversion from laparoscopic surgery to open surgery were not significantly different between the two groups. In addition, the conversion rate in patients who had a history of upper abdominal surgery was similar to other studies in the literature [10-12]. There is no consensus in the literature regarding conversion rates in patients who underwent previous upper abdominal surgery. While a group of studies has suggested that previous abdominal operations did not pose a risk for conversion, because adhesions resulting from these operations did not change the anatomy of the right upper quadrant and therefore did not adversely affect the success of LC, another group of studies has indicated that previous upper abdominal surgeries may indeed be a risk factor for conversion of LC to open surgery [3,8,13-15]. We believe that future studies will lead to a consensus on this issue. We also anticipate that with the increase in laparoscopy experience among surgeons, previous upper abdominal surgery will no longer remain a risk factor for conversion.

In our study, among the patients who had previous upper abdominal surgery, one (2.3%) patient developed a bile duct injury due to intense adhesions. This injury was repaired with surgical intervention (primary repair and T-tube drainage) during the same session. Atasoy et al. have reported the rate of bile duct injury to be 5.5% in patients who had previous upper abdominal surgery [16]. One of the patients with a history of upper abdominal surgery in our study developed a wound infection after open surgery. This infection was controlled with surgical debridement and medical treatment.

Bile duct injury developed in two patients without a history of upper abdominal surgery. While in one patient the surgery was performed during the same session, in the other case, the injury was managed in the next session. Among the same group of patients, one patient developed wound infection, which was controlled with debridement and medical treatment. Seetahal et al. have suggested that previous abdominal surgery increases the risk of iatrogenic bowel injury by causing adhesions or by obstructing the visualization of hepatobiliary structures and limiting the field of view [17]. However, this complication did not occur in any of the patients included in our study. We also did not find any difference between the groups in terms of early complications. However, there are opposing views in the literature regarding this issue. A study by Ercan et al., which included 677 patients who had undergone abdominal surgery, reported results similar to ours; however, Atasoy et al. reported that early complications were higher in patients who had a history of upper abdominal surgery [8,16].

Postoperative complications can prolong the hospital stay in LC, which is one of the most common general surgeries. In our study, no difference was found between the groups in terms of length of hospital stay. We think that similar early complication rates in both groups may explain the similarity in hospital stay between the groups. Given that most of the previous studies have reported higher mean hospital stay in patients who had a history of upper abdominal surgery, we think that the results of our study can bring a new perspective to the literature on this subject [8,18]. In our study, although patients who had previous upper abdominal surgery had more adhesions, similar rates of conversion from laparoscopic to open surgery, early complication rates, and hospital stay between the groups may be due to the advanced laparoscopic surgical experience and well-defined safe cholecystectomy methods with improved laparoscopic surgical instruments.

Our study has some limitations. Firstly, it was a retrospective analysis with some inherent limitations. The small sample size of the group that had a history of upper abdominal surgery can be considered as another limitation. We believe that despite all these limitations, our results shed significant light on the topic and will guide prospective studies to be planned on the subject.

Minimally invasive surgery and applications to accelerate the recovery after surgery are becoming more common throughout the world, including in Turkey. As these practices that are seen as the gold standard in today's surgery become more widespread, the surgeons’ experience will increase with simultaneous technological advancement and the limitations of these applications are in need of a review. It seems that unless there is a specific contraindication for the patient, minimally invasive surgery will become the primary tool of surgeons in the near future.

## Conclusions

Based on our findings, intra-abdominal adhesions and operation time were longer in patients who had a history of upper abdominal surgery, whereas the rate of conversion to open surgery, early complications, and length of hospital stay were similar between both groups of patients. Therefore, we think that LC can be safely applied in patients who have had upper abdominal surgery. However, it should be kept in mind that intra-abdominal adhesions are more common in this group of patients.
